# A Novel Approach to Managing “Double Diabetes” in a Pediatric Patient on Insulin Pump Therapy: An Off-Label Use of a Fixed-Ratio Insulin Degludec/Liraglutide Combination

**DOI:** 10.7759/cureus.114782

**Published:** 2026-08-19

**Authors:** Batool Almoallem, Nandu K Thalange

**Affiliations:** 1 Pediatric Endocrinology, AlJalila Children's Speciality Hospital, Dubai, ARE; 2 Pediatric Endocrinology, Mohammed Bin Rashid University of Medicine and Health Sciences, Dubai, ARE

**Keywords:** case report, double diabetes, glp-1 receptor agonist, insulin pump, insulin resistance, liraglutide, pediatric, type 1 diabetes

## Abstract

The convergence of type 1 diabetes (T1D) and features of type 2 diabetes, such as obesity and insulin resistance, has given rise to the concept of "double diabetes" (DD). This condition poses significant management challenges in the pediatric population, as escalating insulin requirements may exacerbate weight gain and insulin resistance, creating a vicious cycle. Glucagon-like peptide-1 receptor agonists (GLP-1 RAs) are not approved for pediatric T1D but represent a potential therapeutic avenue. We present the case of an 11-year-old girl with antibody-positive T1D on an insulin pump who developed significant features of DD, including obesity, acanthosis nigricans, and escalating insulin requirements that exceeded her pump reservoir capacity. To address both insulin deficiency and severe insulin resistance, a novel off-label approach was initiated using a fixed-ratio combination of basal insulin degludec and the GLP-1 RA liraglutide (IDegLira), administered once daily as an adjunct to her continued insulin pump therapy for bolus insulin delivery. Over an eight-month follow-up period, the patient demonstrated modest weight loss (−2.2 kg) and a reduction in BMI z-score, with a marked improvement in glycemic control. Her mean glucose decreased from 196 mg/dL to 139 mg/dL, and her glucose management indicator improved from 8.0% to 6.6%. Time spent in severe hyperglycemia (>250 mg/dL) was reduced from 26% to 15%. The adjunctive therapy reduced the total daily insulin dose delivered via the pump. This case report highlights the potential utility of a pragmatic, off-label approach that combines a fixed-ratio GLP-1 RA with basal insulin to manage the challenges of pediatric DD. It addressed insulin resistance, improved glycemic control, and mitigated the burdensome cycle of weight gain and escalating insulin doses. This report underscores the need for further research into tailored, multifaceted therapeutic strategies for this growing patient population.

## Introduction

The rising global prevalence of pediatric obesity has intersected with the increasing incidence of type 1 diabetes (T1D), leading to a subset of young patients who present with features of both T1D and type 2 diabetes (T2D) [1,2]. This phenotype, often termed "double diabetes" (DD), is characterized by autoimmune-mediated beta-cell destruction alongside significant insulin resistance [3,4]. The T1D Exchange Clinic Registry in the United States has reported that over one-third of adolescents with T1D are overweight or obese, highlighting the scale of this clinical challenge [5].

Managing DD in children is complex. Standard management of T1D with intensive insulin therapy can itself exacerbate weight gain, which in turn worsens insulin resistance and further increases insulin requirements, creating a vicious cycle [6]. This can lead to a high total daily insulin dose (TDD), sometimes exceeding the capacity of insulin pump reservoirs and necessitating frequent, costly refills. Furthermore, insulin resistance, often clinically indicated by acanthosis nigricans, is associated with a higher risk of both microvascular and macrovascular complications [7,8].

Glucagon-like peptide-1 receptor agonists (GLP-1 RAs) are approved for T2D and obesity and address several key pathophysiological components of DD. In T1D, they suppress glucagon secretion and delay gastric emptying, thereby promoting satiety, weight loss, and improved insulin sensitivity [9]. While not approved for use in pediatric T1D, several studies in adults with T1D, particularly those who are overweight, have shown that adjunctive GLP-1 RA therapy can reduce HbA1c, body weight, and total insulin dose [10-12]. However, data on the pediatric T1D and DD population remain scarce [13].

Our case report describes a novel, off-label therapeutic strategy in an 11-year-old girl with DD on insulin pump therapy who was struggling with severe insulin resistance and escalating insulin needs. We utilized a fixed-ratio combination of a long-acting basal insulin (insulin degludec) and a GLP-1 RA (liraglutide) as an adjunct to her pump therapy to simultaneously address both her insulin deficiency and her profound insulin resistance.

## Case presentation

We report the case of an 11-year-old girl with a four-year history of T1D, diagnosed at age 7. The diagnosis was confirmed by the presence of three positive diabetes-associated autoantibodies (glutamic acid decarboxylase, islet antigen-2, and zinc transporter 8) and a low C-peptide level. Her initial management involved multiple daily injections, followed by initiation of an insulin pump (Omnipod DASH® system, Insulet Corporation, Acton, MA, USA) using a rapid-acting insulin analog (insulin lispro-aabc, Lyumjev®, Eli Lilly and Company, Indianapolis, IN, USA).

Over the two years following her transition to an insulin pump, she experienced significant weight gain with a BMI at the 98th percentile for her age and sex. Clinically, she developed prominent acanthosis nigricans on her neck and axillae, a cutaneous marker of insulin resistance. Her glycemic control deteriorated, with a glucose management indicator (GMI) of 8.0%. Her insulin requirements steadily increased, with her TDD frequently exceeding 80-90 units (approximately 1.3 units/kg/day). This high insulin requirement exceeded the capacity of her Omnipod® reservoir, forcing her to replace the device every 36-48 hours rather than the standard 72 hours. The escalating insulin doses appeared to be fueling a vicious cycle of further weight gain and worsening insulin resistance. This disrupted her daily life and significantly increased the cost of her care.

Given the complex clinical picture of DD, a decision was made to introduce an adjunctive therapy to directly target insulin resistance. The patient was started on a once-daily subcutaneous injection of a fixed-ratio combination of insulin degludec and liraglutide (IDegLira 100/3.6, Xultophy®, Novo Nordisk A/S, Bagsværd, Denmark). The starting dose was 20 dose steps, delivering 20 units of insulin degludec and 0.72 mg of liraglutide. The basal insulin delivery from her insulin pump was correspondingly reduced by 20 units. The bolus insulin for meals and corrections continued to be administered via her insulin pump. The primary goals of this novel, off-label intervention were to improve insulin sensitivity, reduce the TDD, mitigate weight gain, and allow for the full 72-hour wear time of her insulin pump. Efficacy was assessed by monitoring changes in her weight, BMI, TDD, and continuous glucose monitoring (CGM) metrics over the subsequent months.

The introduction of adjunctive IDegLira therapy resulted in marked improvements in both metabolic parameters and practical aspects of the patient's diabetes management over an eight-month follow-up period. The patient's weight stabilized, with a modest 2.2 kg reduction, and her BMI decreased from 29.97 kg/m² to 27.95 kg/m². Importantly, her height remained appropriate, with an increase of 3.1 cm, indicating that weight stabilization was not at the expense of normal growth.

There was a marked improvement in glycemic control, as evidenced by CGM data and a reduction in the severity of acanthosis nigricans. The mean sensor glucose level decreased by 57 mg/dL, and the GMI, an estimate of HbA1c, improved from 8.0% to 6.6%. The time spent in the target glucose range (70-180 mg/dL) increased, while the time spent in significant hyperglycemia (>250 mg/dL) was nearly halved. The patient and her family reported no major or unexpected adverse effects from the new medication, and the single daily injection was well-tolerated. She did report a few episodes of symptomatic hypoglycemia, including mild dizziness; therefore, the insulin pump doses were adjusted and reduced accordingly, and none of the hypoglycemic episodes necessitated further dose reduction or discontinuation of IDegLira. However, she experienced no gastrointestinal adverse effects, and no episodes of acute pancreatitis occurred.

A key outcome was the reduction in TDD from approximately 1.3 units per kg per day to 0.9 units per kg per day. The adjunctive therapy led to a significant reduction in the amount of insulin required from the pump, resolving the issue of premature pump reservoir depletion. The patient was subsequently able to consistently use each insulin pump for its intended 72-hour duration. A summary of the key outcomes is presented in Table 1.

**Table 1:** Clinical and glycemic metrics before and after intervention BMI: body mass index, CGM: continuous glucose monitoring, GMI: glucose management indicator, TDD: total daily insulin dose

Metric	Baseline (pre-intervention)	8-month follow-up (post-intervention)	Change
Anthropometrics			
Weight (kg)	73.3	71.1	-2.2
Height (cm)	156.4	159.5	+3.1
BMI (kg/m²)	29.97	27.95	-2.02
BMI (z-score)	+2.16	+1.97	-0.19
Glycemic control (CGM)			
Mean glucose (mg/dL)	196	139	-57
GMI (%)	8.0	6.6	-1.4
Time in range (70-180 mg/dL) (%)	48	57	+9
Time above 250 mg/dL (%)	26	15	-11
Time below 54 mg/dL (%)	1	4	+3
TDD (units/kg/day)	1.3	0.9	-0.4

## Discussion

This case report details the successful use of a novel, off-label strategy employing a fixed-ratio combination of basal insulin and a GLP-1 RA to manage a complex case of pediatric DD. Our patient exhibited the classical features of this difficult phenotype: autoimmune T1D with superimposed, severe insulin resistance, leading to a burdensome and self-perpetuating cycle of high insulin doses and weight gain [6]. The use of a GLP-1 RA successfully broke this cycle, leading to improvements in glycemic control, weight stabilization, and a substantial reduction in treatment burden.

The concept of DD, first described in 1991, has become increasingly relevant with the global rise in pediatric obesity [3]. Youth with DD occupy a difficult middle ground between T1D and T2D. As shown in a recent study, these patients often remain fully insulin-dependent yet share the metabolic derangements of insulin resistance with youth who have T2D [4]. This highlights the inadequacy of a therapeutic approach focused solely on insulin replacement. Our case underscores the need for a tailored approach that addresses both aspects of the disease pathophysiology.

GLP-1 RAs are a rational choice for targeting the insulin resistance component of DD. Their pleiotropic incretin-based effects, including promoting satiety, slowing gastric emptying, and improving insulin sensitivity, are well-documented in T2D [9]. While their use in T1D is still considered off-label, a growing body of evidence from adult trials supports their role as an adjunctive therapy. Meta-analyses and large trials such as ADJUNCT ONE and Lira-1 have consistently shown that adding liraglutide to insulin therapy in adults with T1D, particularly those with excess weight, results in modest but significant reductions in HbA1c, body weight, and insulin requirements [10-12]. More recent real-world data on newer agents such as semaglutide and tirzepatide in adults with T1D show even more pronounced weight loss and glycemic benefits [14].

However, the application of this evidence to pediatric practice is limited. As a recent review by Shenker and Shalitin highlights, there is a lack of large, randomized controlled trials of GLP-1 RAs in children and adolescents with T1D [13]. Our case, therefore, provides important real-world evidence for this specific population. The use of a fixed-ratio combination product (IDegLira) was a pragmatic choice. It simplified the regimen by providing both a modern basal insulin and a GLP-1 RA in a single daily injection, potentially improving adherence compared to initiating two separate injections. This approach allowed us to reduce the basal insulin delivered by the pump, thereby directly addressing reservoir volume limitations, while the liraglutide component targeted the underlying insulin resistance.

The observed improvements in our patient are consistent with the mechanisms of GLP-1 RA action. The reductions in mean glucose and GMI, along with decreased TDD and clinical improvement in acanthosis nigricans, collectively indicate enhanced insulin sensitivity. However, this interpretation is indirect, as insulin sensitivity was not measured directly. The modest weight reduction, despite ongoing insulin therapy, indicates a disruption of the weight-gain cycle that often affects these patients. The significant reduction in time spent in severe hyperglycemia is particularly noteworthy, as this is a key driver of long-term complication risk.

This case has several limitations. It is a single-patient report, and the follow-up period is limited to eight months. The long-term efficacy and safety of this approach in the pediatric DD population are unknown. Potential side effects of GLP-1 RAs, such as gastrointestinal symptoms or the theoretical risk of pancreatitis, require ongoing monitoring. Nonetheless, this case provides a compelling proof of concept for a novel therapeutic strategy. It suggests that for carefully selected pediatric patients with DD and severe insulin resistance, the off-label use of a GLP-1 RA, particularly in a simplified combination product, can be a highly effective tool.

## Conclusions

This case report illustrates the significant clinical challenge posed by DD in the pediatric population and presents a successful, novel management strategy. The off-label, adjunctive use of a fixed-ratio insulin degludec/liraglutide combination effectively addressed the dual pathophysiology of insulin deficiency and severe insulin resistance. This approach not only led to marked improvements in glycemic control and weight stabilization but also alleviated the significant treatment burden associated with high-dose insulin pump therapy. Our experience suggests that for carefully selected pediatric patients with DD, a personalized approach targeting insulin resistance may be effective. This case highlights the need for prospective, randomized controlled trials to formally evaluate the safety and efficacy of GLP-1 RAs as an adjunctive therapy for this growing and complex patient cohort.
